# A Role of CXCL1 Drives Osteosarcoma Lung Metastasis *via* VCAM-1 Production

**DOI:** 10.3389/fonc.2021.735277

**Published:** 2021-10-25

**Authors:** Chiang-Wen Lee, Yao-Chang Chiang, Pei-An Yu, Kuo-Ti Peng, Miao-Ching Chi, Ming-Hsueh Lee, Mei-Ling Fang, Kuan-Han Lee, Lee-Fen Hsu, Ju-Fang Liu

**Affiliations:** ^1^ Department of Orthopaedic Surgery, Chang Gung Memorial Hospital, Puzi, Taiwan; ^2^ Department of Nursing, Chang Gung University of Science and Technology, Puzi, Taiwan; ^3^ Division of Basic Medical Sciences, and Chronic Diseases and Health Promotion Research Center, Chang Gung University of Science and Technology, Puzi, Taiwan; ^4^ Sports Medicine Center, Chang Gung Memorial Hospital at Chia Yi, Chiayi, Taiwan; ^5^ College of Medicine, Chang Gung University, Taoyuan, Taiwan; ^6^ Department of Respiratory Care, Chang Gung University of Science and Technology, Puzi, Taiwan; ^7^ Division of Pulmonary and Critical Care Medicine, Chang Gung Memorial Hospital, Kaohsiung, Taiwan; ^8^ Division of Neurosurgery, Department of Surgery, Chang Gung Memorial Hospital, Chiayi, Taiwan; ^9^ Center for Environmental Toxin and Emerging-Contaminant Research, Cheng Shiu University, Kaohsiung, Taiwan; ^10^ Super Micro Research and Technology Center, Cheng Shiu University, Kaohsiung, Taiwan; ^11^ Department of Pharmacy, Chia Nan University of Pharmacy and Science, Tainan, Taiwan; ^12^ School of Oral Hygiene, College of Oral Medicine, Taipei Medical University, Taipei, Taiwan; ^13^ Department of Medical Research, China Medical University Hospital, China Medical University, Taichung, Taiwan

**Keywords:** osteosarcoma, metastasis, CXCL1, VCAM-1, migration

## Abstract

Osteosarcoma, a common aggressive and malignant cancer, appears in the musculoskeletal system among young adults. The major cause of mortality in osteosarcoma was the recurrence of lung metastases. However, the molecular mechanisms of metastasis involved in osteosarcomas remain unclear. Recently, CXCL1 and CXCR2 have been crucial indicators for lung metastasis in osteosarcoma by paracrine releases, suggesting the involvement of directing neutrophils into tumor microenvironment. In this study, overexpression of CXCL1 has a positive correlation with the migratory and invasive activities in osteosarcoma cell lines. Furthermore, the signaling pathway, CXCR2/FAK/PI3K/Akt, is activated through CXCL1 by promoting vascular cell adhesion molecule 1 (VCAM-1) *via* upregulation of nuclear factor-kappa B (NF-κB) expression and nuclear translocation. The *in vivo* animal model further demonstrated that CXCL1 serves as a critical promoter in osteosarcoma metastasis to the lung. The correlated expression of CXCL1 and VCAM-1 was observed in the immunohistochemistry staining from human osteosarcoma specimens. Our findings demonstrate the cascade mechanism regulating the network in lung metastasis osteosarcoma, therefore indicating that the CXCL1/CXCR2 pathway is a worthwhile candidate to further develop treatment schemas.

## Introduction

Among all bone malignancy diagnoses with children and young adults, osteosarcoma is the most common primary and aggressive malignant neoplasm (1, 2). Surgical excision, neoadjuvant, and adjuvant chemotherapies are widely used to treat osteosarcoma for the preservation of limb functions and increase of the successful rate of treatment (3); in addition, less than 70% of patients remain alive after 3 years with no clinical metastasis at first diagnosis (4). Osteosarcoma has high potentials of invasion and metastasis; subsequently, osteosarcoma metastases in the lung are the main reason for mortality rate (5). Approximately one-third of the patients presenting with localized disease will relapse, and patients with metastases at diagnosis are nearly three-quarters; approximately 90% of relapses occur because of lung metastases (6). In order to prevent osteosarcoma lung metastasis, effective therapy is urgently required.

Chemokines are low-molecular-weight proteins that have diverse roles in human physiology including cellular development, migration, immune surveillance, inflammation, and various pathological conditions (7). Chemokines are critical for mediating leukocyte trafficking and positioning, stimulating endothelial cell migration, and activating signaling pathways (8). Moreover, chemokines also regulate progression, metastasis, apoptosis, and chemoresistance in cancer cells (9–12). The chemokine (C-X-C motif) ligand 1 (CXCL1) has been linked to the effects on inflammation, angiogenesis, tumorigenesis, and wound healing (13). CXCR2, receptor of CXCL1, is highly expressed on the surface of neutrophils (14) and is involved in progression in several kinds of cancer, such as lung, prostate, breast, colorectal, ovarian, and pancreatic cancer (15). CXCL1 could mediate several kinds of CXCR2-expressed cells, such as neutrophils, macrophages, osteoblasts, and fibroblasts, to regulate the tumor microenvironment (16–18). Besides, the positive correlation of CXCL1 levels depends on the size of tumor, stage of advancing, and depth of invasion while negative to survival rate of patients has been reported (19–21). CXCL1 secretion could enhance the invasion and metastasis of several types of cancer cells (22–25); in contrast, silencing or knockdown of CXCL1 could inhibit tumor growth in hepatocellular carcinoma (26) and colorectal cancer liver metastasis (27). These studies conspicuously indicate that CXCL1 is a crucial indicator in the progress of cancer invasion and metastasis. However, the role and related mechanisms of CXCL1 in osteosarcoma remain unclear.

Vascular cell adhesion molecule 1 (VCAM-1/CD106), an inducible surface glycoprotein, belongs to the immunoglobulin superfamily (28). VCAM-1 expressions influence numerous biological activities in normal conditions including the regulation of migration and invasion during the cancer metastasis (28). Overexpression of VCAM-1 could occur in the metastatic cancer cells and correlated with the stage of disease including tumor progression in osteosarcoma enhancing the connective tissue growth factor stimulation to promote migration and metastasis (9, 29, 30).

The current study showed that the CXCL1 could signal the CXCR2/focal adhesion kinase (FAK)/phosphatidylinositol-3-kinase (PI3K)/Akt/nuclear factor-kappa B (NF-κB) pathway to increase VCAM-1 expression and subsequently upregulate the metastasis ability of human osteosarcoma cells. The strategy to suppress the expression level of CXCL1 could effectively decrease the VCAM-1 expression, which has the beneficial response for inhibiting migration, invasion, and wound healing abilities in osteosarcoma. This finding strongly suggests that CXCL1/CXCR2 signals act as indicators in metastatic osteosarcoma and could be a therapeutic target for developing anticancer medicine with their unique characteristics.

## Materials and Methods

### Materials

All chemical reagents were purchased from Sigma-Aldrich (St. Louis, MO, USA). The CXCL1, CXCR2, VCAM-1, p-FAK, FAK, p-p85α, p85α, p-Akt, Akt, p-IKKα/β, IKKα/β, p-IκBα, IκBα, p-p65, p65, and β-Actin specific anti-mouse and anti-rabbit IgG-conjugated horseradish peroxidase antibodies were purchased from GeneTex International Corporation (Hsinchu City, Taiwan). Recombinant human CXCL1 was purchased from PeproTech (Rocky Hill, NJ, USA). Short hairpin RNA (shRNA) plasmid for CXCR2 and VCAM-1 were obtained from the National RNAi Core Facility Platform (Taipei, Taiwan). All siRNAs were ON-TARGETplus siRNAs, obtained from Dharmacon Research (Lafayette, CO, USA). The NF-κB luciferase report plasmid, pSV-β-galactosidase vector, and luciferase assay kit were purchased from Promega (Madison, WI, USA).

### Cell Culture

The normal osteoblast cell lines (hFOB1.19) and human osteosarcoma cell lines (MG63, U2OS, and HOS) were purchased from the American Type Cell Culture Collection (Manassas, VA, USA). The hFOB1.19 cells were cultured in DMEM/F12 medium, U2OS cells were cultured in McCoy’s 5A medium, and MG63 and HOS cells were cultured in Eagle’s minimum essential medium. All cell mediums were supplemented with 20 mM HEPES, 10% heat-inactivated fetal bovine serum, and 2 mM-glutamine. Cells were maintained at 37°C in a humidified atmosphere of 5% CO_2_/95% air. Migration-prone MG63 cells were selected by their differential migration abilities as described previously (9).

### Establishment of Migration-Prone Subclones From Osteosarcoma Cell Line

The MG63 (M10 and M20) migration-prone subclones were established by using Transwell inserts. MG63 osteosarcoma cells (1 × 10^6^) suspended in 1 ml of serum-free medium were seeded in the upper chamber, while 1 ml of growth medium contained 10% FBS was loaded into the lower compartment. After 24 h, the cells that migrated across the Transwell insert to the bottom of plate were detached by trypsin and cultured as MG63 (M1). The cells were cultured for 2 days for a second round of selection. The MG63 migration-prone subclone continued migration selection for 10 and 20 rounds to generate MG63 (M10) and MG63 (M20), respectively.

### Immunoblotting Assay

Total cell lysates were collected by using RIPA lysis buffer for further immunoblotting assay. Afterward, equal amounts of the protein were separated by SDS-polyacrylamide gel electrophoresis and transferred to polyvinyl difluoride (PVDF) membranes. Blots were blocked with 5% nonfat milk for at room temperature for 1 h; moreover, blots were incubated for another 1 h with different primary antibodies at room temperature to measure the levels of the targets. The peroxidase conjugated secondary antibody (1:5,000) at room temperature for 1 h after three washes to clear residue of primary antibodies. The signals were detected on a charge-coupled device camera-based detection system (UVP Inc., Upland, CA, USA), and the ImageJ software (National Institutes of Health, USA) was used for quantifying. At least three independent immunoblotting datasets were collected from the experiments and a closer figure pattern was selected for presentation.

### RNA Extraction and Quantitative Real-Time PCR

Total RNA was isolated from cells by using Total RNA preparation kits (easy-Blue Total RNA Extraction kit, iNtRON Biotechnology, Seongnam, Korea) following the manufacturer’s protocol. The RNA was reverse transcribed to cDNA by reverse transcriptase (Invitrogen, Carlsbad, CA, USA). Quantitative real-time PCR (qPCR) was used to determine the mRNA levels of target genes by running on a StepOnePlus machine (Applied Biosystems, Foster City, CA, USA). The SYBR Green fluorescence probe system (KAPA Biosystems, Woburn, MA, USA) was used for determining the threshold cycle (C_T_) of target genes. Primers of human VCAM-1 and glyceraldehyde 3-phosphate dehydrogenase (GAPDH) were purchased from Sigma-Aldrich. The expression levels of the target genes were normalized to GAPDH levels and the formula of level ratio = 2^−ΔΔCt^, where ΔΔCt = (Ct _target_−Ct _GADPH_)_Sample_−(Ct _target_−Ct _GADPH_)_Control_, was used for calculation. The data were represented with three independent experiments with triplicate of each sample.

### Transwell Cell Migration Assay

The Transwell inserts (8-μm pore size; Costar, NY, USA) in 24-well dishes were used for cell migration assay. Cells (2 × 10^4^ cell/well) were pretreated with the designated inhibitors for 90 min and then incubated for 24 h in the culture supernatants. The cells were seeded in the upper Transwell chamber, and 300 μl of medium was prepared into the lower chamber. Cells were fixed in 3.7% formaldehyde for 30 min and stained with 0.05% crystal violet for 60 min after 24-h incubation was finished. Each chamber was washed with PBS after removing upper side cells by cotton-tipped swabs. The cells located on the underside of the filter were examined and counted under a microscope. The data were collected from at least three independent experiments.

### Immunofluorescence Microscopy

MG63 cells (5 × 10^3^ cell/well) were seeded on glass coverslips and treated with designed conditions. Once PBS was rinsed, the cells on the slice were fixed in 3.7% paraformaldehyde at room temperature for 15 min. Then, cells were washed three times with PBS to remove the residual of the fixed solution and then 4% BSA was used for blocking with another 15 min. The cells were incubated with anti-human p65 (1:100) at room temperature for 1 h. The cells were further incubated with FITC-conjugated goat anti-rabbit IgG for 1 h after twice PBS washed. Leica TCS SP2 Spectral Confocal System was used for photographing the mounted cells.

### Reporter Assay

Cells (2 × 10^5^ cell/well) were co-transfected with NF-κB report plasmid and pSV-β-galactosidase vector for 24 h by using Lipofectamine 3000™ (Invitrogen). Cells were lysed with lysis buffer (100 μl) and harvested by centrifugation (13,200 rpm for 15 min) for further luciferase assay. The supernatants with 1:4 luciferase assay buffer were reacted. Activities of luciferase were measured by a microplate luminometer and normalized to β-galactosidase expression vector co-transfection efficiency.

### Chromatin Immunoprecipitation Assay

Details of chromatin immunoprecipitation (ChIP) analysis were described previously (9). In brief, DNA samples were immunoprecipitated by the anti-p65 antibody. The immunoprecipitated DNA after phenol-chloroform purification was further amplified with PCR and separated by 1.5% agarose gel electrophoresis. The signals were visualized and photographed by ultraviolet illumination. The promoter region of the NF-κB region (−2167 and −1967) in the promoter region of human VCAM-1 was amplified with primers 5’-ACAGAGAGAGGAGCTTCAGCAGTGAGAGCA-3’ and 5’-GTCTGTGCTTTATAAAGGGTCTTGTTGCAG-3’.

### CXCL1 Knockdown in Osteosarcoma Cell Lines

The lentiviral expression system for CXCL1 knockdown was purchased from the National RNAi Core Facility (RNAi Core, Academia Sinica, Taiwan). The CXCL1 shRNA plasmid was selected to knock down gene expression. The osteosarcoma cell line MG63 was transfected with the CXCL1 shRNA plasmid. The cells were puromycin-selected and the surviving cells were used as stable gene-modified cell lines. MG63 cell lines that stably expressed luciferase were established before transfection with the CXCL1 shRNA vector or the control vector, and the *in vivo* orthotopic model was analyzed using the *In Vivo* Imaging Systems (IVIS, Xenogen, UK).

### Orthotopic Animal Model and Imaging

Ethical approval was obtained for the use of the animals, and all experiments were performed in accordance with the Guidelines for Animal Care of the Institutional Animal Care and Use Committee of College of Medicine, National Taiwan University (Approval No: 20150357). CB17/SCID mice were purchased from Lasco Inc. (Taipei, Taiwan) weighing 20–30 g, and were acclimatized to a room maintained at 25°C and 50% ± 10% humidity under a 12-h day–night cycle for at least 3 days before experimentation. Individual mice were anesthetized with isoflurane (1%–3%) and oxygen (100%) inhalation. The cortex of the tibial crest was penetrated using a 27-gauge needle, 10 μl containing 1×10^6^ cells were injected. Four weeks after injection, the tumor growth and local metastasis were monitored using IVIS Imaging System. The mice were euthanized by CO_2_ inhalation. The lung tissues were removed and fixed in 10% formalin for further analysis. The number of lung tumor nodules was counted under a dissecting microscope. The experiment was repeated twice.

### Histological Analysis of Lung Metastases

All of the lungs resected from mice were fixed with 10% buffered formalin and embedded in paraffin. Thick sections of 7 μm were stained with hematoxylin and eosin by using histological analyses.

### Immunohistochemistry Staining

Human osteosarcoma tissue arrays (consisting 11 cases of normal bone, 7 cases of stage I osteosarcoma, 49 cases of stage II osteosarcoma, and 7 cases of stage III osteosarcoma) were applied for investigating the expression level of CXCL1 and VCAM-1. The clinical specimens were rehydrated and incubated in 3% hydrogen peroxide to block endogenous peroxidase activity. The slices after antigen retrieval were incubated in 3% bovine serum albumin and then the primary mouse polyclonal anti-CXCL1 and VCAM-1 antibodies (1:100 dilution) at 4°C overnight. After thrice PBS washes, slides were incubated with biotin-labeled goat anti-mouse IgG secondary antibody (1:100 dilution) and signals were amplified with the ABC Kit (Vector Laboratories, Burlingame, CA, USA). Sections on slides were stained with the chromogen diaminobenzidine, washed, counterstained with Delafield’s hematoxylin, dehydrated, and treated with xylene, and then the bound signals were mounted and photographed by microscope. Intensities of tumor cell staining were scored from 0 to 5, where 0 = no staining or unspecific staining, 1 = very weak (intensity), 2 = weak staining, 3 = moderate staining, 4 = strong staining, and 5 = very strong staining. A pathologist scored staining intensity in all samples.

### Statistical Analysis

Data were expressed as mean ± standard deviation (SD). Student’s *t*-test and one-way ANOVA, followed by post-hoc Fisher LSD multiple comparisons (multiple groups), were used. The coefficient of determination (*r* ^2^) was used to evaluate the performance of a linear regression model and for modeling the coefficient of signals. A *p*-value < 0.05 was considered significant.

## Results

### CXCL1 Stimulates Migration, Wound Healing, and Invasion in Osteosarcoma Cells

**Figure 1:**
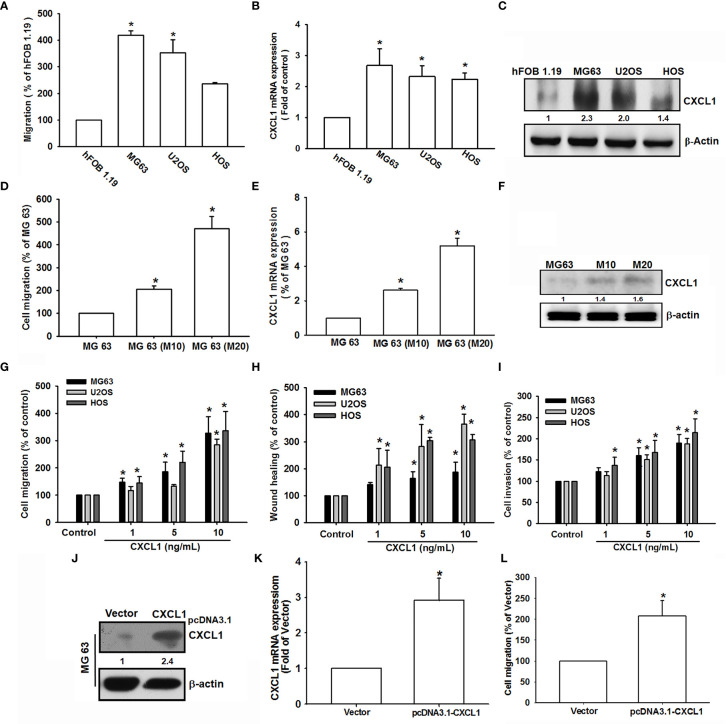
CXCL1 increased migration, invasion, and wound healing in human osteosarcoma MG63, U2OS, and HOS cells. **(A)** The cell migration ability of the osteoblast cell line hFOB 1.19 and the osteosarcoma cell lines MG63, U2OS, and HOS was assessed using the Transwell assay. **(B, C)** Total mRNA and protein were collected from the indicated cell lines, and CXCL1 expression was detected using qPCR and Western blotting. **(D–F)** Migratory ability and CXCL1 expression of the indicated cells (MG63, M10, and M20) were examined by Transwell, qPCR, and immunoblotting assays. **(G–I)** Cells were incubated with CXCL1 (1–10 ng/ml) and the Transwell assay were used for detecting *in vitro* migratory, wound healing, and invasive activities after 18 h. **(J–L)** Cells were transfected with pcDNA3.1-conjugated CXCL1 plasmid and then CXCL1 mRNA, protein expression, and migratory potential were measured by immunoblotting, qPCR, and Transwell assays. Results were expressed as mean ± S.D. from at least three individual experiments. *p < 0.05 compared with the control group of each experiment.

CXCL1 has been integrated to migration and metastasis in various types of cancer cells (21, 25, 26). To select the suitable human osteosarcoma cell lines (MG-63, HOS, and U2OS) and to further investigate the role of CXCL1 on invasion and metastases, the migration ability was measured. As shown in **Figure 1A**, MG63 has a rapid cell migration ability compared to other osteosarcoma cell lines; furthermore, the CXCL1 expression levels were also consistent with the trend of migration ability (**Figures 1B, C**).

The previously established high-migration-prone sublines, MG-63 (M10) and MG-63 (M20) (31), were also used to test the correlation of CXCL1 expression and migratory activity. Results showed that migration-prone sublines, MG-63 (M20) and MG-63 (M10), had a better cell motility, CXCL1 mRNA, and protein expression than the original MG63 cell line (**Figures 1D** **–F**). These findings implied a relationship of CXCL1 expression and metastasis in osteosarcoma cells. Next, CXCL1 (1–10 ng/ml) was applied to investigate whether CXCL1 expression influences cell motility in these three osteosarcoma cell lines. Results suggested that a concentration-dependently facilitated migration, wound healing, and invasive abilities could be affected in all osteosarcoma cell lines (**Figures 1G** **–I**). Furthermore, the pCDNA3.1-conjugated CXCL1 plasmid was transfected into MG63 cells to evaluate the relationship of CXCL1 levels and cell migration. **Figures 1J, K** indicated that the CXCL1 mRNA and protein have enhanced the expression level and the increases of migratory ability were also observed (**Figure 1L**) after transfected with the pCDNA3.1-CXCL1 plasmid. According to these results, the expression of CXCL1 showed a positive regulation on the migratory, wound healing, and invasive abilities in osteosarcoma cells.

### CXCL1 Stimulates VCAM-1 Expression in Osteosarcoma Cells

**Figure 2:**
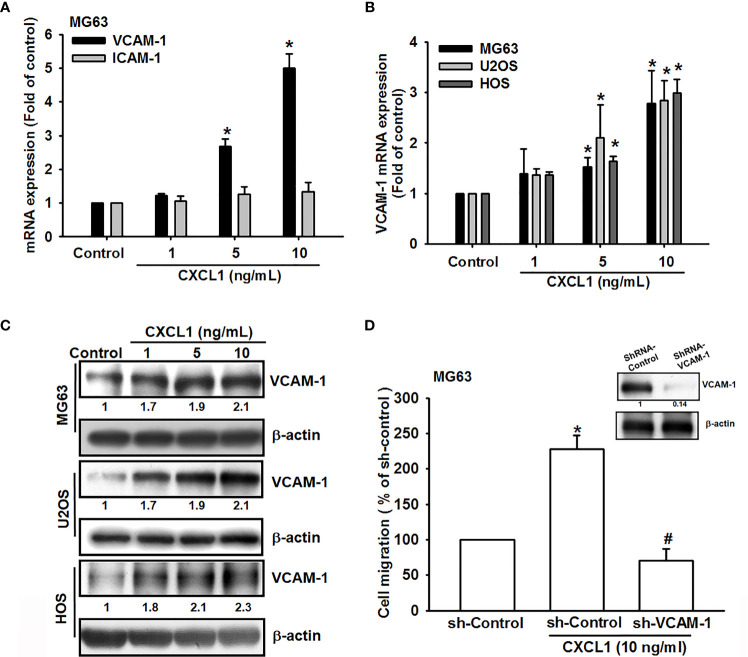
CXCL1 promotes osteosarcoma migration by increasing VCAM-1 expression. **(A)** MG63 cells were stimulated with CXCL1 (1–10 ng/ml) and the mRNA levels of VCAM-1 and ICAM-1 were examined by real-time qPCR. **(B, C)** CXCL1 increased the VCAM-1 mRNA and protein expression in MG63, U2OS, and HOS human osteosarcoma cells. **(D)** VCAM-1 expression and migratory potential were examined by immunoblotting and Transwell assays in MG63 cells, which were transfected with VCAM-1 shRNA for 24 h and then stimulated with CXCL1 for another 24 h. The data were collected from at least three individual experiments and expressed as mean ± S.D. *p < 0.05 as compared to the control group; ^#^ *p* < 0.05 compared with the CXCL1-treated shRNA-control group.

Cell adhesion molecules play critical roles during the extravasation step, a situation where cancer cells adhere to the vasculature endothelium of small capillaries and then migrate through the vasculature wall to generate metastatic foci of tumor metastasis (32), but few known in human osteosarcoma. Previous studies suggested that VCAM-1-dependent motility is an essential factor for tumor metastatic developments (33, 34). To investigate tumor metastatic developments, the role of VCAM-1 in CXCL1 upon osteosarcoma cell migration was examined. Results (**Figure 2A**) showed that CXCL1 (1–10 ng/ml) could increase the VCAM-1 expression in a dose-dependent manner in MG63 cell, but no effects on intercellular adhesion molecule-1 (ICAM-1). Furthermore, the mRNA and protein levels of VCAM-1 were upregulated after CXCL1 stimulation (**Figures 2B, C**). Cells with VCAM-1 shRNA transfection could suppress CXCL1-induced VCAM-1 protein expressions and migration in MG63 osteosarcoma cells (**Figure 2D**). These results indicated that CXCL1 facilitated VCAM-1-dependent cell migration in osteosarcoma.

### CXCL1 Stimulates VCAM-1 Expression and Cell Migration *via* CXCR2 Receptor in Osteosarcoma Cells

**Figure 3:**
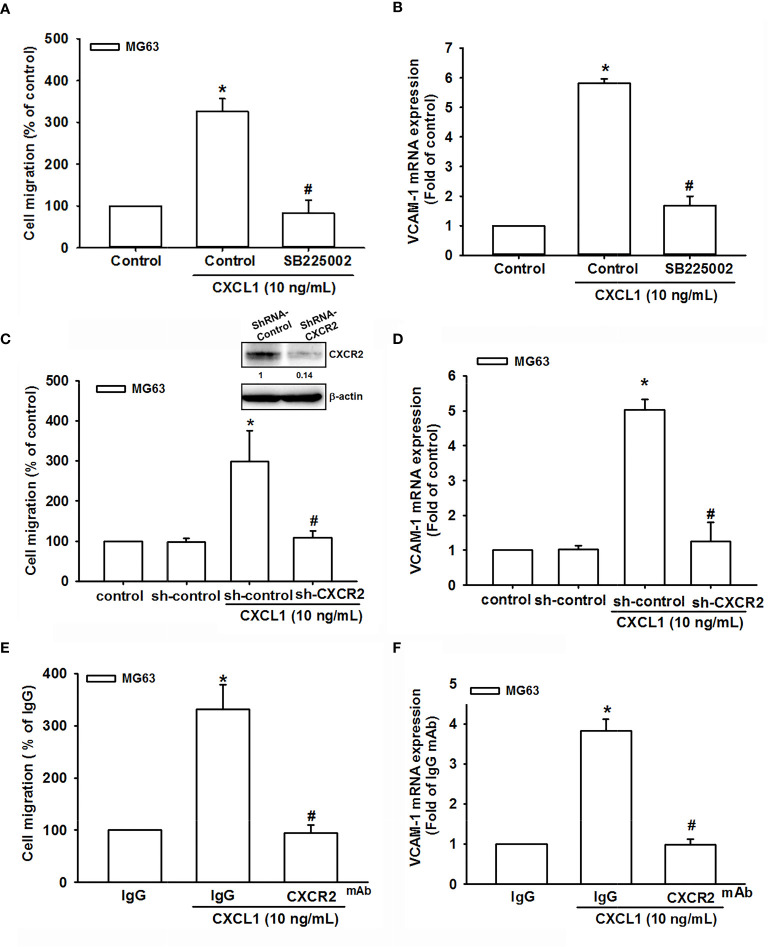
The role of the CXCR2 receptor in CXCL1-mediated cell migration and VCAM-1 expression. **(A, B)** The CXCR2 antagonist, SB225002. **(C, D)** CXCR2-shRNA transfection. **(E, F)** CXCR2 neutralizing antibody reduced CXCL1-induced cell migration and VCAM-1 expression in MG63 cells. The data were collected from at least three individual experiments and expressed as mean ± S.D. *p < 0.05 as compared to the control group; ^#^ *p* < 0.05 compared with the CXCL1-treated shRNA-control and IgG groups.

The CXCR2 is a specific receptor for CXCL1 and is involved in CXCL1-mediated cancer progress (9, 15, 17). To further investigate whether CXCL1-induced VCAM-1 expression and cell migration are CXCR2-dependent mechanisms in osteosarcoma, the antagonist (SB225002), CXCR2 shRNA, and neutralizing antibody of CXCR2 were used for evaluation. As shown in **Figures 3A, B**, CXCR2 antagonist-SB225002 could significantly suppress CXCL1 (10 ng/ml)-induced cell migration and mRNA expression of VCAM-1. Similar results were observed in CXCR2 shRNA (**Figures 3C, D**) and CXCR2 neutralizing antibody (**Figures 3E, F**). These results demonstrated that CXCL1-mediated cell migration and VCAM-1 expression were upregulated by binding to the CXCR2 receptor.

### The FAK/PI_3_K/AKT/NF-κB Signaling Pathway Is Essential for CXCL1-Induced Increases in VCAM-1 Expression and Cell Migration

**Figure 4:**
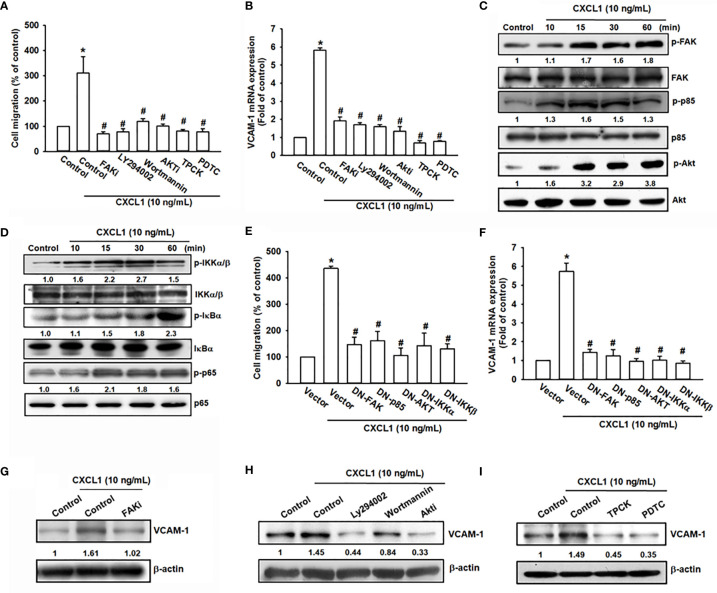
The FAK, PI3K, Akt, and NF-κB pathways are involved in CXCL1-promoted migration and VCAM-1 expression in MG63 osteosarcoma cell. **(A, B)** CXCL1-increased cell migration and VCAM-1 mRNA expression could be reduced by pre-treated FAK inhibitor (2 μM), PI3K inhibitors LY294002 (5 μM) and wortmannin (2 μM), Akt inhibitor (5 μM), and NF-κB inhibitors TPCK (5 μM) and PDTC (1 μM) for 90 min. **(C)** Phosphorylated levels of FAK, P85, and Akt, and **(D)** phosphorylated levels of IKK, IκBα, and p65 were examined by immunoblotting assay in MG-63 cells with CXCL1 incubation for the indicated time intervals (0, 10, 15, 30, or 60 min). **(E, F)** Transfecting dominant-negative mutants of FAK, PI3K, Akt, and IKK for 24 h could suppress CXCL1 increased cell migration and VCAM-1 mRNA expression. **(G–I)** FAK, PI3K, Akt, and NF-κB inhibitors reduced CXCL1-induced VCAM-1 protein expressions. The data were collected from at least three individual experiments and expressed as mean ± S.D. *p < 0.05 as compared to the control group; ^#^ *p* < 0.05 compared with the CXCL1-treated control or vector group.

CXCL1/CXCR2-mediating signaling cascades, such as PI3K/Akt, mitogen-activated protein kinase (MAPK), and nuclear factor kappa-light-chain-enhancer of activated B cells (NF-κB), have been considered the regulatory signaling pathways for migration and invasion in several cancer cells (15, 35). Additionally, a previous study also suggested that CXCL8 is activated through the CXCR2 receptor to stimulate the downstream FAK, c-Src protein tyrosine kinase activity (36). To investigate the potential downstream signaling pathway by CXCL1 activation of CXCR2, the candidate signals of FAK/PI3K/Akt were screened by different inhibitors. Inhibitors of FAK (FAKi), PI3K (LY294002 and wortmannin), Akt (Akti), and NF-κB (TPCK and PDTC) were applied and showed a clear suppression pattern of CXCL1-triggered cell migration and VCAM-1 mRNA expression in MG63 osteosarcoma cells (**Figures 4A, B**). Furthermore, the phosphorylation of FAK, PI3K, Akt, and NF-κB were upregulated by CXCL1 treatment (**Figures 4C, D**). Dominant-negative mutants of several molecules in FAK/PI3K/Akt/NF-κB abolished CXCL1-promoted MG63 cell migration and VCAM-1 mRNA expression (**Figures 4E, F**). Moreover, CXCL1-enhanced VCAM-1 protein levels could be suppressed by pretreating respective pathway inhibitors (**Figures 4G** **–I**). These findings indicated that CXCL1-triggered cell migration and VCAM-1 expression were regulated through the FAK/PI3K/Akt/NF-κB pathway in osteosarcoma cells.

### The NF-κB Signals Are Involved in CXCL1-Promoted Cell Migration and VCAM-1 Expression

**Figure 5:**
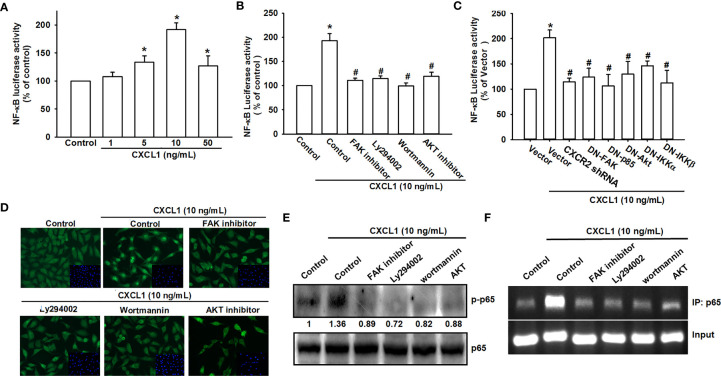
NF-κB mediates the response of human osteosarcoma cells by CXCL1 stimulation. **(A)** The luciferase activity of NF-κB promoter plasmid was incubated with the indicated doses of CXCL1 for 24 h in MG63 cells. CXCL1 (1-50 ng/ml)-stimulated NF-κB luciferase activity was suppressed by **(B)** inhibitors of FAK, PI3K, and AKT and **(C)** dominant negative FAK, PI3K, AKT, IKK, or CXCR2 shRNA. **(D)** The anti-p65 and DAPI signals by pretreated several inhibitors and then CXCL1 (10 ng/ml)-stimulated, representative confocal microscopy images were shown. **(E)** CXCL1-induced p65 phosphorylation and **(F)** chromatin immunoprecipitation of anti-p65 in nuclear extracts of MG63 cells were measured with pretreated inhibitors. One percent of immunoprecipitated chromatin was assayed to verify equal loading (input). Results are expressed as the mean ± S.D. of triplicate samples. *p < 0.05 as compared to the control group; ^#^ *p* < 0.05 compared with the CXCL1-treated control or vector group.

NF-κB has been demonstrated as a crucial factor for cancer cell migration and invasion (33, 37, 38). The NF-κB luciferase promoter plasmid was used for evaluating the possibility of CXCL1-mediated CXCR2/FAK/PI3K/Akt pathway on NF-κB expression. As shown in **Figure 5A**, CXCL1 could enhance the luciferase activity of the NF-κB promoter as a reversed U pattern and the peak located at 10 ng/ml. CXCL1-induced NF-κB promoter luciferase activity could be suppressed by CXCR2/FAK/PI3K/Akt/NF-κB pathway inhibitors, dominant-negative mutants, and shRNA (**Figures 5B, C**). Expression level of phosphorylated p65 and nuclear translocation with FAK, PI3K, and Akt inhibitors pretreatment were measured to confirm the signaling transduction cascade. The inhibitors of FAK, PI3K, and Akt reversed CXCL1-induced p65 phosphorylation and nuclear translocation (**Figures 5D, E**). In addition, transcriptional activation of NF-κB was further investigated whether it participates in CXCL1-promoted VCAM-1 expression. The chromatin immunoprecipitation (ChIP) assay was examined for evaluation and it was suggested that these pathway inhibitors could abolish the binding ability of CXCL1-induced p65 to the NF-κB binding element on the VCAM-1 promoter (**Figure 5F**). These results demonstrated that the CXCL1-driven CXCR2/FAK/PI3K/Akt pathway is involved in the regulation of NF-κB expression and nuclear translocation, and subsequently affected the expression of NF-κB-dependent VCAM-1.

### Suppression of CXCL1 Expression in Osteosarcoma Reduces Metastatic Colonies Arising in Pulmonary Vasculature *In Vivo*

**Figure 6:**
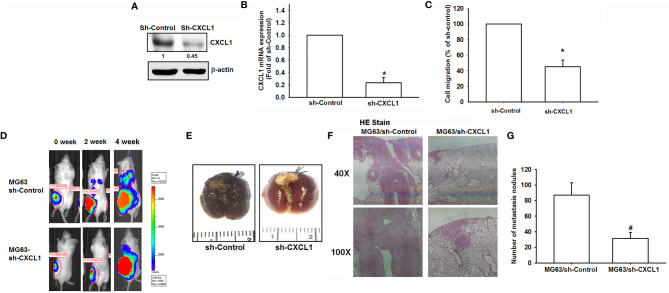
The CXCL1/CXCR2 axis is required for osteosarcoma lung metastasis. **(A, B)** Protein and mRNA levels of CXCL1 and **(C)** cell migration ability of MG63 transfected with sh-CXCLl by immunoblotting, real-time qPCR, and Transwell assays in MG63 cells. **(D)** The mice were injected with MG63/control shRNA, or MG63/CXCL1 shRNA cells. Lung metastasis was monitored by bioluminescence imaging at the indicated time intervals. **(E–G)** The appearance of hematoxylin and eosin stain and numbers of metastasis nodules of lung specimens from sacrifice mice after 4 weeks of cell injection was shown. Results are expressed as the mean ± S.D. of triplicate samples. */^#^ *p* < 0.05 as compared to the shRNA-control group..

To confirm findings that CXCL1 is a positive regulator for osteosarcoma metastasis to the lung, an *in vivo* animal study was conducted. The transfection of CXCL1 shRNA could significantly reduce the CXCL1 protein, mRNA expression (**Figures 6A, B**), and cell migration ability of MG63 cells (**Figure 6C**).

An orthotopic mouse model was developed for osteosarcoma. MG63 cells were transduced with luciferase and clonally selected for orthotopic implantation and then orthotopically implanted into the right leg tibia. After 4 weeks, IVIS findings showed that the knockdown of CXCL1 reduced tumor metastasis to the lung (**Figure 6D**). Furthermore, the appearance, histochemistry, and number of metastasis nodules were significantly reduced in the lung specimens from sacrifice sh-CXCL1 mice as compared with their control group (**Figures 6E** **–G**). These results clearly suggested that the CXCL1 is a key regulator for osteosarcoma metastasis.

### CXCL1 and VCAM-1 Levels Are Positively Correlated in Human Osteosarcoma Tissue

**Figure 7:**
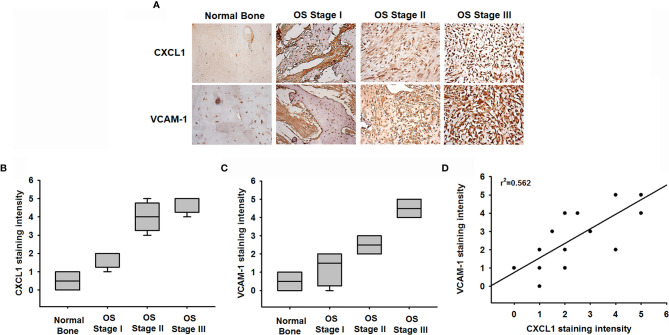
Clinical significance of CXCL1 and VCAM-1 in osteosarcoma patient specimens. **(A)** CXCL1 and VCAM-1 expression levels in specimens were determined in a tumor tissue microarray by IHC staining. The stained specimens were photographed using an optical microscope. **(B, C)** IHC stain intensities were scored from 1 to 5 to quantify CXCL1 and VCAM-1 expression levels in different stages. **(D)** Correlation between CXCL1 and VCAM-1 expression in human osteosarcoma specimens. Results are shown as mean ± S.D.

We next examined levels of CXCL1 and VCAM-1 expression in osteosarcoma specimens, to determine the prognostic relevance of CXCL1 and VCAM-1 in osteosarcoma progression. The IHC results revealed higher levels of CXCL1 and VCAM-1 expression in patients with a higher-grade osteosarcoma than in those with a lower-grade osteosarcoma; the levels of CXCL1 and VCAM-1 expression were reflected by the tumor stage (**Figures 7A** **–C**). These results illustrate how the levels of CXCL1 and VCAM-1 expression were significantly higher in higher-stage tumors than in lower-stage tumors. A positive correlation observed between the CXCL1 and VCAM-1 staining intensity of the human osteosarcoma tissue (*r* ^2^ = 0.562, **Figure 7D**) indicated that the levels of these proteins were associated with the progression of osteosarcoma.

## Discussion

Osteosarcoma is a common primary but aggressive with high metastatic potential cancer in the musculoskeletal system of childhood and adolescence (1, 2). The high chemoresistance development and lung metastasis propensities of osteosarcoma imply that developing an effective adjuvant therapy for inhibiting osteosarcoma metastasis is an important and urgent issue for treating osteosarcoma. Chemokines have pro-inflammatory functions and play various roles in regulations of cell activation, differentiation, adhesion, and trafficking in immune cells involved in reprogramming of somatic cells to pluripotent stem cells (39–41). Moreover, upregulations of chemokines to facilitate proliferation and manage apoptosis, survival, and metastasis in several types of cancer are observed (10, 33). Recently, evidence suggests that CXCL1 secreted from tumor through the paracrine or autocrine mechanisms attracted various inflammatory cells (10, 21, 32, 42) or stromal cells (43, 44) into the tumoral microenvironment promoting tumor growth and metastasis, such as bladder (16), lung (17), and liver metastasis colorectal cancer (42). CXCL1 could directly recruit circulatory CXCR2-expressing neutrophils and MDSCs into inflammatory sites, developing a supportive metastatic microenvironment as pre-metastatic niches (15–18, 21). A recent study suggested that CXCL1 secreted from human pulmonary artery endothelial cells played a homing role for osteosarcoma metastasis to the lung (9). In the current study, our *in vitro* and *in vivo* studies showed that CXCL1 directly facilitates cell migration and invasion abilities, and metastasis nodule formation to the lung in osteosarcoma. The result of the study firmly indicates that the CXCL1/CXCR2 is an essential axis for metastasis of osteosarcoma.

Overexpression of FAK mediating PI3K/Akt and MEK-extracellular signal-regulated kinase 1/2 (ERK1/2) signal transductions contributes to the antiapoptotic property for cancer survival (45). It is well known that the PI3K/Akt pathway is a critical regulatory signaling for almost all human cancers (46). PI3K is a heterodimer consisting of a catalytic subunit (p110) and an adaptor/regulatory subunit (p85) that could be activated by receptors with protein tyrosine kinase or G protein-coupled receptors and activated to its downstream Akt signals (47). The PI3K/Akt pathway has been approved and associated with cell-death regulation (such as suppression of Bad proapoptotic or caspase-9 induced apoptotic activities; inducing of NF-κB transcriptional activity), cell cycle progression, and cell growth (such as modulation of mTOR activity and inhibition of GSK3 catalytic activity) (47). Findings from Kinome profiling and genome-wide gene expression data showed a notable phenomenon in that most osteosarcoma cell lines existed with active Akt signals (48). This indicated that the PI3K/Akt-mediated pathways also play important roles in osteosarcoma. Moreover, serine/threonine kinases including the PI3K/Akt and MAPK signaling cascades have been suggested, which could be activated by CXCR2 receptor (36, 49). Integration of previous studies and our findings shows that dysregulation of CXCL1/CXCR2 and their downstream PI3K/Akt pathways is implicated in the metastasis progresses of osteosarcoma (invasion, migration, etc.) including several pathological progresses (tumorigenesis, proliferation, angiogenesis, and chemoresistance).

In this study, NF-κB expression and nuclear translocation could be raised by activating the PI3K/Akt pathway. NF-κB is an essential transcription factor that could bind the 5’-regulatory region of VCAM-1 promoting expression level of VCAM-1 (9, 50). According to the current results, all NF-κB inhibitors suppressed CXCL1-induced VCAM-1 mRNA, protein expressions, and migratory and invasive activities of MG63 cells, indicating that the activation of NF-κB is essential for these metastases’ progress. Furthermore, CXCL1 facilitates NF-κB luciferase activity and enhances NF-κB p65 subunit to bind to the NF-κB binding site on the VCAM-1 promoter region. The CXCL1-increased activities of NF-κB upon VCAM-1 expression could be suppressed by inhibitors of CXCR2, FAK, PI3K, and Akt, indicating that these expressions are regulated by the CXCR2/FAK/PI3K/Akt pathway.

To establish the disseminated cancer cells that may finally cause metastases, the cancer cells were released by primary tumors into the circulation system at an early stage to adapt to new environmental stress, and transmigration of cells across endothelial capillary walls is the initial step (51). Transendothelial migration was the followed step, which occurred in monocytes or leukocytes that extravasate to the endothelial cell wall at inner blood vessels of the underlying tissues (52). VCAM-1 has been supported to associate with early leukocyte transmigration to the circulation system and into the extravasation to underlying tissues (52). Moreover, interactions of VCAM-1 with stroma are key factors for survival of metastatic cancer cells. A previous study has shown that lung metastatic breast cancer cells aberrantly expressed ICAM-1- and VCAM-1-promoted metastatic cells for survival in the parenchyma microenvironment of lung (53). Although ICAM-1 has also been shown to play roles during the initiation of the metastatic cascade driving tumor progression (54), this development seems to not involve the CXCL1-triggered metastatic cascade in osteosarcoma cells. Our results suggested that CXCL1 *via* the CXCR2/FAK/PI3K/Akt/NF-κB pathway enhanced VCAM-1 expression to assist the migratory and invasive activity in osteosarcoma cells. Evidence demonstrates that VCAM-1 advocated cascade relevance for tumorigenesis and metastasis and may be associated with the survival of osteosarcoma metastatic cells.

In conclusion, the current study demonstrates that chemokine CXCL1 plays a critical promotive factor *via* the CXCR2/FAK/PI3K/Akt pathway to upregulate the NF-κB expression and nuclear translocation, and further triggered the NF-κB-dependent VCAM-1 expression enhancing the migration, invasion, and homing abilities of osteosarcoma cells to the lung. Our investigation suggests that CXCL1/CXCR2, as a therapeutic candidate, can be targeted to further develop medicine to suppress or prevent the metastatic spread of osteosarcoma.

## Data Availability Statement

The raw data supporting the conclusions of this article will be made available by the authors, without undue reservation.

## Ethics Statement

Ethical approval was obtained for the use of the animals, and all experiments were performed in accordance with the Guidelines for Animal Care of the Institutional Animal Care and Use Committee of College of Medicine, National Taiwan University (Approval No. 20150357).

## Author Contributions

J-FL, Y-CC, C-WL, and L-FH conceived and designed the experiments, which were performed by J-FL, L-FH, P-AY, M-CC, and K-TP. M-HL, M-LF, K-HL, and C-WL analyzed the data. C-WL, Y-CC, M-CC, K-TP, M-HL, and M-LF contributed reagents/materials/analysis tools. J-FL, L-FH, C-WL, and Y-CC wrote the paper. All authors contributed to the article and approved the submitted version.

## Funding

This study was supported by grants from the Ministry of Science and Technology, Taiwan, R.O.C. (MOST 109-2320-B-255-004-MY3, MOST106-2314-B-038-099-MY3) and Taipei Medical University (TMU108-AE1-B47), and by Chang Gung Medical Research Program Foundation, grant number CMRPF6K0041, CMRPF6J0051, CMRPF6J0052 and CMRPF6K0021. Chang Gung University of Science and Technology (rants ZRRPF3K0111).

## Conflict of Interest

The authors declare that the research was conducted in the absence of any commercial or financial relationships that could be construed as a potential conflict of interest.

## Publisher’s Note

All claims expressed in this article are solely those of the authors and do not necessarily represent those of their affiliated organizations, or those of the publisher, the editors and the reviewers. Any product that may be evaluated in this article, or claim that may be made by its manufacturer, is not guaranteed or endorsed by the publisher.

## References

[1] Ottaviani G Jaffe N. The Etiology of Osteosarcoma. Cancer Treat Res (2009) 152:15–32. doi: 10.1007/978-1-4419-0284-9_2 20213384

[2] Arndt CA Crist WM. Common Musculoskeletal Tumors of Childhood and Adolescence. N Engl J Med (1999) 341(5):342–52. doi: 10.1056/NEJM199907293410507 10423470

[3] Fan TM Roberts RD Lizardo MM. Understanding and Modeling Metastasis Biology to Improve Therapeutic Strategies for Combating Osteosarcoma Progression. Front Oncol (2020) 10:13. doi: 10.3389/fonc.2020.00013 32082995 PMC7006476

[4] Isakoff MS Bielack SS Meltzer P Gorlick R. Osteosarcoma: Current Treatment and a Collaborative Pathway to Success. J Clin Oncol (2015) 33(27):3029–35. doi: 10.1200/JCO.2014.59.4895 PMC4979196 26304877

[5] Ando K Mori K Verrecchia F Marc B Redini F Heymann D. Molecular Alterations Associated With Osteosarcoma Development. Sarcoma (2012) 2012:523432. doi: 10.1155/2012/523432 22448123 PMC3289857

[6] Briccoli A Rocca M Salone M Guzzardella GA Balladelli A Bacci G. High Grade Osteosarcoma of the Extremities Metastatic to the Lung: Long-Term Results in 323 Patients Treated Combining Surgery and Chemotherapy, 1985-2005. Surg Oncol (2010) 19(4):193–9. doi: 10.1016/j.suronc.2009.05.002 19515554

[7] Raman D Sobolik-Delmaire T Richmond A. Chemokines in Health and Disease. Exp Cell Res (2011) 317(5):575–89. doi: 10.1016/j.yexcr.2011.01.005 PMC3063402 21223965

[8] Sarvaiya PJ Guo D Ulasov I Gabikian P Lesniak MS. Chemokines in Tumor Progression and Metastasis. Oncotarget (2013) 4(12):2171–85. doi: 10.18632/oncotarget.1426 PMC3926818 24259307

[9] Chao CC Lee CW Chang TM Chen PC Liu JF. CXCL1/CXCR2 Paracrine Axis Contributes to Lung Metastasis in Osteosarcoma. Cancers (Basel) (2020) 12(2):459–75. doi: 10.3390/cancers12020459 PMC7072404 32079335

[10] Do HTT Lee CH Cho J. Chemokines and Their Receptors: Multifaceted Roles in Cancer Progression and Potential Value as Cancer Prognostic Markers. Cancers (Basel) (2020) 12(2):287–12. doi: 10.3390/cancers12020287 PMC7072521 31991604

[11] Lien MY Tsai HC Chang AC Tsai MH Hua CH Wang SW. Chemokine CCL4 Induces Vascular Endothelial Growth Factor C Expression and Lymphangiogenesis by miR-195-3p in Oral Squamous Cell Carcinoma. Front Immunol (2018) 9:412. doi: 10.3389/fimmu.2018.00412 29599774 PMC5863517

[12] Liu GT Huang YL Tzeng HE Tsai CH Wang SW Tang CH. CCL5 Promotes Vascular Endothelial Growth Factor Expression and Induces Angiogenesis by Down-Regulating miR-199a in Human Chondrosarcoma Cells. Cancer Lett (2015) 357(2):476–87. doi: 10.1016/j.canlet.2014.11.015 25444917

[13] Dhawan P Richmond A. Role of CXCL1 in Tumorigenesis of Melanoma. J Leukoc Biol (2002) 72(1):9–18. doi: 10.1189/jlb.72.1.9 12101257 PMC2668262

[14] Eash KJ Greenbaum AM Gopalan PK Link DC. CXCR2 and CXCR4 Antagonistically Regulate Neutrophil Trafficking From Murine Bone Marrow. J Clin Invest (2010) 120(7):2423–31. doi: 10.1172/JCI41649 PMC2898597 20516641

[15] Jaffer T Ma D. The Emerging Role of Chemokine Receptor CXCR2 in Cancer Progression. Trans Cancer Res (2016) 5(Suppl 4):S616–28. doi: 10.21037/tcr.2016.10.06

[16] Miyake M Hori S Morizawa Y Tatsumi Y Nakai Y Anai S. CXCL1-Mediated Interaction of Cancer Cells With Tumor-Associated Macrophages and Cancer-Associated Fibroblasts Promotes Tumor Progression in Human Bladder Cancer. Neoplasia (2016) 18(10):636–46. doi: 10.1016/j.neo.2016.08.002 PMC5043399 27690238

[17] Yuan M Zhu H Xu J Zheng Y Cao X Liu Q. Tumor-Derived CXCL1 Promotes Lung Cancer Growth *via* Recruitment of Tumor-Associated Neutrophils. J Immunol Res (2016) 2016:6530410. doi: 10.1155/2016/6530410 27446967 PMC4942661

[18] Hardaway AL Herroon MK Rajagurubandara E Podgorski I. Marrow Adipocyte-Derived CXCL1 and CXCL2 Contribute to Osteolysis in Metastatic Prostate Cancer. Clin Exp Metastasis (2015) 32(4):353–68. doi: 10.1007/s10585-015-9714-5 PMC4393805 25802102

[19] Ogata H Sekikawa A Yamagishi H Ichikawa K Tomita S Imura J. GROalpha Promotes Invasion of Colorectal Cancer Cells. Oncol Rep (2010) 24(6):1479–86. doi: 10.3892/or_00001008 21042742

[20] Xiang Z Jiang DP Xia GG Wei ZW Chen W He Y. CXCL1 Expression Is Correlated With Snail Expression and Affects the Prognosis of Patients With Gastric Cancer. Oncol Lett (2015) 10(4):2458–64. doi: 10.3892/ol.2015.3614 PMC4579984 26622871

[21] Li L Xu L Yan J Zhen ZJ Ji Y Liu CQ. CXCR2-CXCL1 Axis Is Correlated With Neutrophil Infiltration and Predicts a Poor Prognosis in Hepatocellular Carcinoma. J Exp Clin Cancer Res (2015) 34:129. doi: 10.1186/s13046-015-0247-1 26503598 PMC4621872

[22] Acharyya S Oskarsson T Vanharanta S Malladi S Kim J Morris PG. A CXCL1 Paracrine Network Links Cancer Chemoresistance and Metastasis. Cell (2012) 150(1):165–78. doi: 10.1016/j.cell.2012.04.042 PMC3528019 22770218

[23] Xu J Zhang C He Y Wu H Wang Z Song W. Lymphatic Endothelial Cell-Secreted CXCL1 Stimulates Lymphangiogenesis and Metastasis of Gastric Cancer. Int J Cancer (2012) 130(4):787–97. doi: 10.1002/ijc.26035 21387301

[24] Cheng WL Wang CS Huang YH Tsai MM Liang Y Lin KH. Overexpression of CXCL1 and Its Receptor CXCR2 Promote Tumor Invasion in Gastric Cancer. Ann Oncol (2011) 22(10):2267–76. doi: 10.1093/annonc/mdq739 21343381

[25] Kawanishi H Matsui Y Ito M Watanabe J Takahashi T Nishizawa K. Secreted CXCL1 Is a Potential Mediator and Marker of the Tumor Invasion of Bladder Cancer. Clin Cancer Res (2008) 14(9):2579–87. doi: 10.1158/1078-0432.CCR-07-1922 18451219

[26] Han KQ He XQ Ma MY Guo XD Zhang XM Chen J. Targeted Silencing of CXCL1 by siRNA Inhibits Tumor Growth and Apoptosis in Hepatocellular Carcinoma. Int J Oncol (2015) 47(6):2131–40. doi: 10.3892/ijo.2015.3203 26499374

[27] Bandapalli OR Ehrmann F Ehemann V Gaida M Macher-Goeppinger S Wente M. Down-Regulation of CXCL1 Inhibits Tumor Growth in Colorectal Liver Metastasis. Cytokine (2012) 57(1):46–53. doi: 10.1016/j.cyto.2011.10.019 22129625

[28] Schlesinger M Bendas G. Vascular Cell Adhesion Molecule-1 (VCAM-1)–an Increasing Insight Into Its Role in Tumorigenicity and Metastasis. Int J Cancer (2015) 136(11):2504–14. doi: 10.1002/ijc.28927 24771582

[29] Kong DH Kim YK Kim MR Jang JH Lee S. Emerging Roles of Vascular Cell Adhesion Molecule-1 (VCAM-1) in Immunological Disorders and Cancer. Int J Mol Sci (2018) 19(4):1057–73. doi: 10.3390/ijms19041057 PMC5979609 29614819

[30] Wang LH Tsai HC Cheng YC Lin CY Huang YL Tsai CH. CTGF Promotes Osteosarcoma Angiogenesis by Regulating miR-543/Angiopoietin 2 Signaling. Cancer Lett (2017) 391:28–37. doi: 10.1016/j.canlet.2017.01.013 28108312

[31] Hou CH Lin FL Hou SM Liu JF. Cyr61 Promotes Epithelial-Mesenchymal Transition and Tumor Metastasis of Osteosarcoma by Raf-1/MEK/ERK/Elk-1/TWIST-1 Signaling Pathway. Mol Cancer (2014) 13:236. doi: 10.1186/1476-4598-13-236 25326651 PMC4210521

[32] Miles FL Pruitt FL van Golen KL Cooper CR. Stepping Out of the Flow: Capillary Extravasation in Cancer Metastasis. Clin Exp Metastasis (2008) 25(4):305–24. doi: 10.1007/s10585-007-9098-2 17906932

[33] Liu JF Lee CW Lin CY Chao CC Chang TM Han CK. CXCL13/CXCR5 Interaction Facilitates VCAM-1-Dependent Migration in Human Osteosarcoma. Int J Mol Sci (2020) 21(17):6095–108. doi: 10.3390/ijms21176095 PMC7504668 32847038

[34] Chang AC Chen PC Lin YF Su CM Liu JF Lin TH. Osteoblast-Secreted WISP-1 Promotes Adherence of Prostate Cancer Cells to Bone *via* the VCAM-1/Integrin Alpha4beta1 System. Cancer Lett (2018) 426:47–56. doi: 10.1016/j.canlet.2018.03.050 29627497

[35] Yang G Rosen DG Liu G Yang F Guo X Xiao X. CXCR2 Promotes Ovarian Cancer Growth Through Dysregulated Cell Cycle, Diminished Apoptosis, and Enhanced Angiogenesis. Clin Cancer Res (2010) 16(15):3875–86. doi: 10.1158/1078-0432.CCR-10-0483 PMC2930833 20505188

[36] Cohen-Hillel E Yron I Meshel T Soria G Attal H Ben-Baruch A. CXCL8-Induced FAK Phosphorylation *via* CXCR1 and CXCR2: Cytoskeleton- and Integrin-Related Mechanisms Converge With FAK Regulatory Pathways in a Receptor-Specific Manner. Cytokine (2006) 33(1):1–16. doi: 10.1016/j.cyto.2005.11.006 16406804

[37] Liu JF Lee CW Tsai MH Tang CH Chen PC Lin LW. Thrombospondin 2 Promotes Tumor Metastasis by Inducing Matrix Metalloproteinase-13 Production in Lung Cancer Cells. Biochem Pharmacol (2018) 155:537–46. doi: 10.1016/j.bcp.2018.07.024 30031810

[38] Tang CH Tan TW Fu WM Yang RS. Involvement of Matrix Metalloproteinase-9 in Stromal Cell-Derived Factor-1/CXCR4 Pathway of Lung Cancer Metastasis. Carcinogenesis (2008) 29(1):35–43. doi: 10.1093/carcin/bgm220 17916907

[39] Lee SJ Kang KW Kim JH Lee BH Jung JH Park Y. CXCR2 Ligands and mTOR Activation Enhance Reprogramming of Human Somatic Cells to Pluripotent Stem Cells. Stem Cells Dev (2020) 29(3):119–32. doi: 10.1089/scd.2019.0188 31808362

[40] Mantovani A Sica A Sozzani S Allavena P Vecchi A Locati M. The Chemokine System in Diverse Forms of Macrophage Activation and Polarization. Trends Immunol (2004) 25(12):677–86. doi: 10.1016/j.it.2004.09.015 15530839

[41] Moser B Willimann K. Chemokines: Role in Inflammation and Immune Surveillance. Ann Rheum Dis (2004) 63(Suppl 2):ii84–9. doi: 10.1136/ard.2004.028316 PMC1766778 15479880

[42] Wang D Sun H Wei J Cen B DuBois RN. CXCL1 Is Critical for Premetastatic Niche Formation and Metastasis in Colorectal Cancer. Cancer Res (2017) 77(13):3655–65. doi: 10.1158/0008-5472.CAN-16-3199 PMC5877403 28455419

[43] Kasashima H Yashiro M Nakamae H Masuda G Kinoshita H Morisaki T. Clinicopathologic Significance of the CXCL1-CXCR2 Axis in the Tumor Microenvironment of Gastric Carcinoma. PloS One (2017) 12(6):e0178635. doi: 10.1371/journal.pone.0178635 28575019 PMC5456266

[44] Zhang T Tseng C Zhang Y Sirin O Corn PG Li-Ning-Tapia EM. CXCL1 Mediates Obesity-Associated Adipose Stromal Cell Trafficking and Function in the Tumour Microenvironment. Nat Commun (2016) 7:11674. doi: 10.1038/ncomms11674 27241286 PMC4895055

[45] Tai YL Chen LC Shen TL. Emerging Roles of Focal Adhesion Kinase in Cancer. BioMed Res Int (2015) 2015:690690. doi: 10.1155/2015/690690 25918719 PMC4396139

[46] Porta C Paglino C Mosca A. Targeting PI3K/Akt/mTOR Signaling in Cancer. Front Oncol (2014) 4:64. doi: 10.3389/fonc.2014.00064 24782981 PMC3995050

[47] Fresno Vara JA Casado E de Castro J Cejas P Belda-Iniesta C Gonzalez-Baron M. PI3K/Akt Signalling Pathway and Cancer. Cancer Treat Rev (2004) 30(2):193–204. doi: 10.1016/j.ctrv.2003.07.007 15023437

[48] Kuijjer ML van den Akker BE Hilhorst R Mommersteeg M Buddingh EP Serra M. Kinome and mRNA Expression Profiling of High-Grade Osteosarcoma Cell Lines Implies Akt Signaling as Possible Target for Therapy. BMC Med Genomics (2014) 7:4. doi: 10.1186/1755-8794-7-4 24447333 PMC3932036

[49] Waugh DJ Wilson C. The Interleukin-8 Pathway in Cancer. Clin Cancer Res (2008) 14(21):6735–41. doi: 10.1158/1078-0432.CCR-07-4843 18980965

[50] Sharma R Sharma R Khaket TP Dutta C Chakraborty B Mukherjee TK. Breast Cancer Metastasis: Putative Therapeutic Role of Vascular Cell Adhesion Molecule-1. Cell Oncol (Dordr) (2017) 40(3):199–208. doi: 10.1007/s13402-017-0324-x 28534212 PMC13001545

[51] Husemann Y Geigl JB Schubert F Musiani P Meyer M Burghart E. Systemic Spread Is an Early Step in Breast Cancer. Cancer Cell (2008) 13(1):58–68. doi: 10.1016/j.ccr.2007.12.003 18167340

[52] Muller WA. Mechanisms of Leukocyte Transendothelial Migration. Annu Rev Pathol (2011) 6:323–44. doi: 10.1146/annurev-pathol-011110-130224 PMC3628537 21073340

[53] Chen Q Zhang XH Massague J. Macrophage Binding to Receptor VCAM-1 Transmits Survival Signals in Breast Cancer Cells That Invade the Lungs. Cancer Cell (2011) 20(4):538–49. doi: 10.1016/j.ccr.2011.08.025 PMC3293160 22014578

[54] Benedicto A Romayor I Arteta B. Role of Liver ICAM-1 in Metastasis. Oncol Lett (2017) 14(4):3883–92. doi: 10.3892/ol.2017.6700 PMC5604125 28943897

